# Crystal structure and Hirshfeld analysis of (1a*S*,3a*R*,4a*S*,5a*R*)-15-acet­oxy­linden-7(11),8-trieno-12,8-lactone

**DOI:** 10.1107/S2056989022004625

**Published:** 2022-05-20

**Authors:** Qiang-Qiang Lu, Xin-Wei Shi, Ya-Fu Zhou, Xin-Ai Cui, Hong Wang

**Affiliations:** a Shaanxi Engineering Research Centre for Conservation and Utilization of Botanical Resources, Xi’an Botanical Garden of Shaanxi Province (Institute of Botany of Shaanxi Province), Xi’an 710061, People’s Republic of China; Venezuelan Institute of Scientific Research, Venezuela

**Keywords:** crystal structure, sesquiterpenes, polycyclic framework, *Chloranthus japonicus*

## Abstract

The structure of the title com­pound, C_17_H_20_O_4_ [systematic name: (1a*S*,3a*R*,4a*S*,5a*R*)-15-(acet­oxy)linden-7(11),8-trieno-12,8-lactone or (4a*R*,5*S*,5a*R*,6a*S*,6b*R*)-5-(acet­oxy­meth­yl)-4a,5,5a,6,6a,6b-hexa­hydro-3,6b-di­methyl­cyclo­propa[2,3]indeno­[5,6-*b*]furan-2(4*H*)-one, *ent*-chloranthalactone C], a natural product iso­lated from the whole plant *Chloranthus japonicus* Sieb., is a typical lin­den­ane-type sesquiterpenoid. The mol­ecule com­prises a bi­cyclo­[3.1.0]hexane ring (*A*/*B* system) bearing an acetoxymethyl (C-4) group, a bi­cyclo­[4.3.0]nonane ring (*B*/*C* system) containing a double bond (C-8/9) and a chiral quaternary carbon (C-10), and a 7(11)-en-12,8-olide structural moiety on the cyclo­hexan-8-ene (*C* ring). In the tetra­cyclic skeleton, the 1,3-cyclo­propane ring has a β-con­figuration, and atoms H-5 and H_3_-14 have α- and β-orientations, respectively. In the crystal, the mol­ecules are assembled into a two-dimensional network by weak O⋯H/H⋯O inter­actions. Hirshfeld surface analysis illustrates that the greatest contributions are from H⋯H (55.2%), O⋯H/H⋯O (34.6%) and C⋯H/H⋯C (8.9%) contacts.

## Abstract 2

The title com­pound, isolated from the *Chloranthus japonicus* Sieb., is a typical lindenane-type sesquiterpenoid. Hirshfeld surface analysis illustrates that the most important contributions are from O⋯H/H⋯O contacts (34.6%).

## 1. Chemical context

Lindenanolides are precursors for various sesquiterpene dimer derivatives (Uchida *et al.*, 1980 ▸; Wang *et al.*, 2009 ▸; Shi *et al.*, 2016 ▸). Inspired by the clinical application of artemisinin, these com­pounds have become a products library for screening anti­malarial drugs (Dondorp *et al.*, 2010 ▸; Zhou *et al.*, 2017 ▸). The roots of *Chloranthus japonicus* (called Yinxiancao) were reported to exhibit anti­fungal and anti-inflammatory activities, and have been used as traditional Chinese medicine to treat malaria (Kawabata & Mizutani, 1989 ▸). Chloran­tha­lactone C was characterized as an α,β,γ,δ-unsaturated γ-lac­tone and was converted into desacetyl enol lactone hydrate and ketoalcohol under moderate alkaline conditions (Uchida *et al.*, 1980 ▸). Because of the unique stereostructure in lindenane, these lactone derivatives have been studied extensively and serve as precursors for screening cytotoxicity against mouse lymphosarcoma, liver cancer and human cervical cancer cells, the expression of cell adhesion mol­ecules and the mode of anti­plasmodial agents (Uchida *et al.*, 1980 ▸; Zhang *et al.*, 2012 ▸; Zhou *et al.*, 2017 ▸). Based on the anti­wiggler activity, we are currently searching for a biological pesticide preparation to inhibit flyblow breeding in vegetable production (Shi *et al.*, 2016 ▸) and report here the structure of the title com­pound.

## 2. Structural commentary

**Figure 1:**
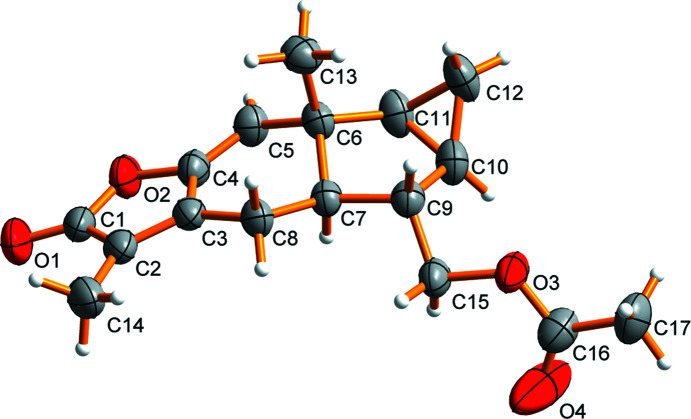
The mol­ecular structure of the title com­pound, showing the atomic numbering scheme. Displacement ellipsoids are drawn at the 50% probability level.

The mol­ecular structure of the title com­pound is shown in Scheme 1 and Fig. 1 ▸. This com­pound consists of a novel polycyclic framework embedded with a sterically congested cyclo­pentane ring (*B*), an unusual *trans*-5/6 ring junction and an angular methyl group. The chiral quaternary C atom at the 10-position is located on the same side of the *B* ring plane as the cyclo­propane ring and the 4-acet­oxy­methyl and 5-hydrogen are positioned on the other side. The positions of the substituents can be described as having a β-configuration for the cyclo­propane ring at the 1,3-positions, axial for the H atom at the 5-position and bis­ectional for the methyl H atom at the chiral quaternary C atom in the 10-position. Two cyclic olefinic bonds are located between atoms C2 and C3, and between atoms C4 and C5, and are attached to the cyclo­hexane (*C*) and cyclo­penta­nolactone (*D*) rings, respectively. The torsion angles C9—C10—C11—C12 and C12—C10—C11—C6 of 115.2 (4) and −115.2 (4)°, respectively, describe the geometric metamerism of the junction between cyclo­propane ring *A* and cyclo­pentane ring *B*. The difference in configuration of the oxygen-containing groups can be confirmed by the torsion angles C7—C9—C15—O3 and O1—C1—O2—C4, which were 179.9 (3) and −179.0 (4)°, respectively. The torsion angles C5—C6—C11—C12 and C2—C3—C8—C7 are the same at 155.5 (4)°, indicating the conformational stability of the *A*/*B* and *C*/*D* ring junctions. Also, the C2—C3—C4—C5 and C8—C3—C4—O2 torsion angles are 177.1 (4) and 177.2 (3)°, respectively, and the O2—C1—C2—C14 and C14—C2—C3—C4 torsion angles are 179.9 (3) and −178.9 (4)°, respectively, and describe the geometric characteristics of the *C* and *D* rings. In the title mol­ecule, the central six-membered lindenane ses­qui­terpenoid ring has a half-chair conformation, with puckering parameters (Cremer & Pople, 1975 ▸; Luger & Bülow, 1983 ▸) of *Q*_T_ = 0.3387 (11) Å, θ = 49.11 (19)° and ψ = 167.3 (2)°. Furthermore, the C9—C7—C8—C3 and C5—C4—O2—C1 torsion angles [−178.6 (3) and −177.6 (4)°, respectively] indicate the geometric stability of the *B*/*C* and *C*/*D* ring junctions. In addition, the main *A*/*B*/*C*/*D* skeleton and the acetoxymethyl system (atoms C15–C17/O3/O4) are not coplanar, the torsion angles C15—O3—C16—C17 and C15—O3—C16—O4 being −175.9 (3) and 2.8 (6)°, respectively. 
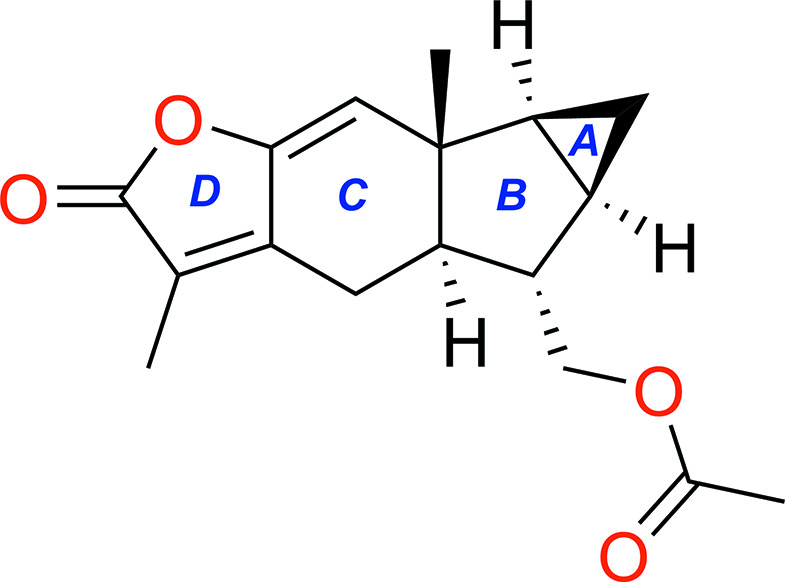


## 3. Supra­molecular features

**Figure 2:**
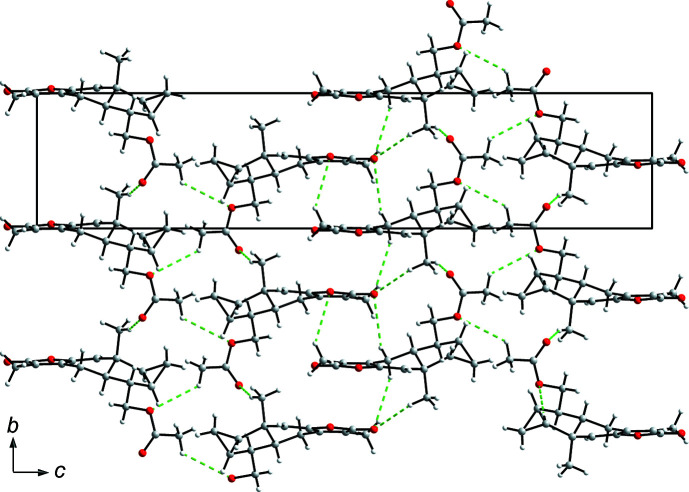
The packing of mol­ecules in the crystal structure of the title com­pound, viewed along the *c* direction (C—H⋯O hydrogen bonds are shown as green dashed lines).

In the crystal of the title com­pound, the mol­ecules are linked *via* multiple C—H⋯O weak hydrogen bonds, generating two-dimensional (2D) layers propagating along the *c*-axis direction (Fig. 2 ▸ and Table 1 ▸). Details of the hydrogen-bonding inter­actions and the symmetry codes are given in Table 1 ▸.

## 4. Hirshfeld surface analysis

**Figure 3:**
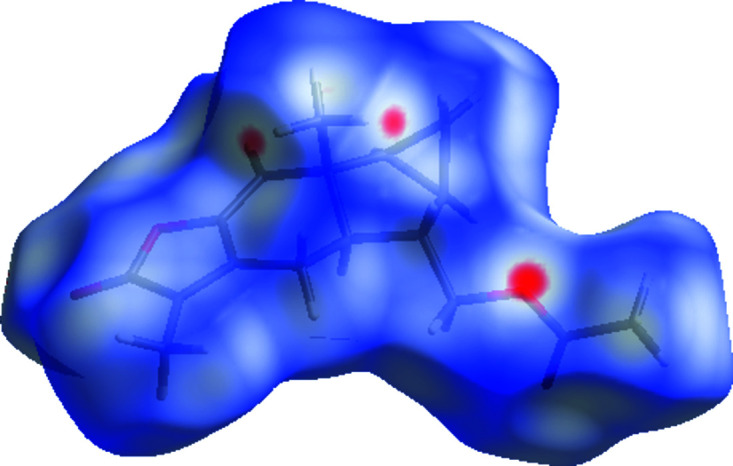
Front view of the 3D Hirshfeld surface of the title com­pound mapped over *d*_norm_ in the range from −0.1253 to 1.4046 arbitrary units.

**Figure 4:**
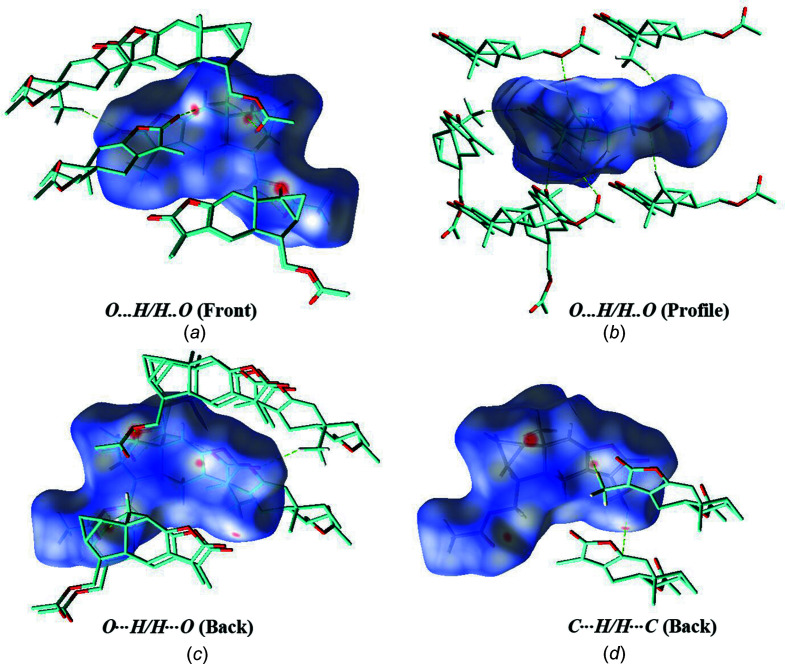
Hirshfeld surface mapped over *d*_norm_ for the mol­ecules of the title com­pound showing: (*a*) H⋯O/O⋯H contacts (front), (*b*) H⋯O/O⋯H contacts (profile), (*c*) H⋯O/O⋯H contacts (back) and (*d*) C⋯H/H⋯C contacts (back). H atoms not involved in bonding have been omitted for clarity.

**Figure 5:**
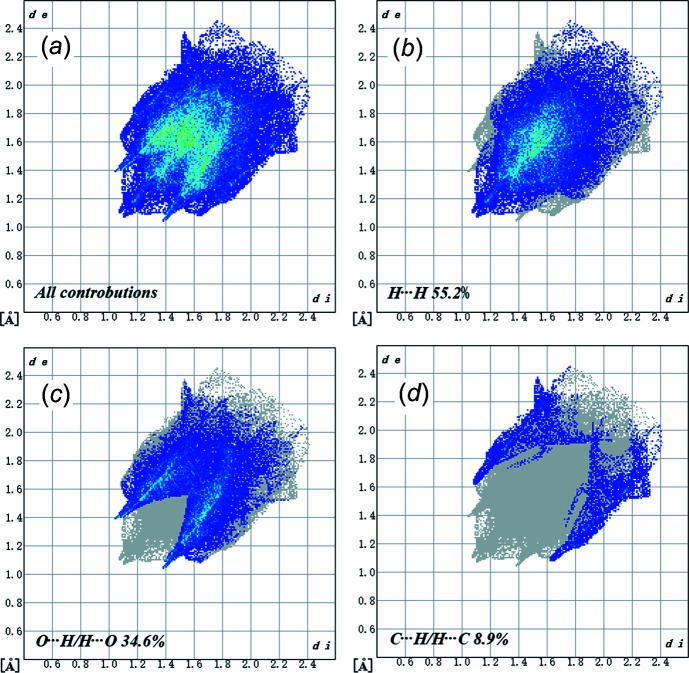
The 2D fingerprint plots of the title com­pound, showing (*a*) all inter­actions, and delineated into (*b*) H⋯H, (*c*) O⋯H/H⋯O and (*d*) C⋯H/H⋯C inter­actions. The *d*_e_ and *d*_i_ values represent the distances (in Å) from a point on the Hirshfeld surface to the nearest atoms inside and outside the surface, respectively.

Hirshfeld surface analysis was performed and the associated fingerprint plots, providing a 2D view of the inter­molecular inter­actions within the mol­ecular crystals, were generated using *CrystalExplorer* (Version 21.5; Spackman *et al.*, 2021 ▸), with a standard resolution of the three-dimensional (3D) *d*_norm_ surfaces plotted over a fixed colour scale of −0.1253 (red) to 1.4046 (blue) arbitrary units (Fig. 3 ▸). The intense red spots symbolize short contacts and negative *d*_norm_ values on the surface are related to the presence of C—H⋯O hydrogen bonds in the crystal structure. This result corresponds to the results obtained from the solid crystalline structure with the formation of hydrogen bonds. Weak C⋯H/H⋯C contacts are shown by dim red spots (Fig. 4 ▸). The 2D fingerprint plots for the H⋯H, H⋯O/O⋯H, and H⋯C/C⋯H contacts are shown in Fig. 5 ▸. H⋯H inter­actions play an integral role in the overall crystal packing, contributing 55.2%, and are located in the middle region of the fingerprint plot. The most significant H⋯O/O⋯H contacts contribute 34.6% to the Hirshfeld surface and the proportion of weak H⋯C/C⋯H contacts is 8.9%.

## 5. Database survey

A search of the Cambridge Structural Database (CSD, Version 5.43, last update November 2021; Groom *et al.*, 2016 ▸) for the same carbon ring skeleton as the title com­pound yielded only one mol­ecule, 5-[(*tert*-butyl­dimethyl­sil­yl)­oxy]-3,6b-dimethyl-4a,5,5a,6,6a,6b-hexa­hydro­cyclo­propa[2,3]indeno­[5,6-*b*]furan-2(4*H*)-one (CCDC reference 804060; Qian & Zhao, 2011 ▸), which has a (*tert*-butyl­dimethyl­sil­yl)­oxy group attached to ring *A* of the carbon skeleton.

## 6. Isolation and crystallization

The title sesquiterpenoid was isolated as a colourless solid from the EtOAc soluble fraction of *C. japonicus* by chromatography over silica gel, and eluted with a mixture of ethyl acetate and hexane (1:20 to 5:1 *v*/*v* gradient) to yield the title com­pound. Crystals were obtained after recrystallization from acetone or chloro­form–methanol (6:1 *v*/*v*) at room temperature by slow evaporation over a period of a few days. ^1^H NMR (500 MHz, chloro­form-*d*): δ 6.22 (1H, *s*, H-9), 4.20 (2H, *d*, *J* = 6.1 Hz, H-11), 2.63 (1H, *d*, *J* = 13.0 Hz), 2.30–2.21 (2H, *m*), 2.09 (3H, *s*, OCOCH_3_), 1.87 (3H, *br s*, H-13), 1.73 (1H, *tt*, *J* = 10.1, 4.9 Hz), 1.53 (1H, *td*, *J* = 8.1, 3.8 Hz), 1.30 (1H, *ddd*, *J* = 11.9, 8.0, 3.7 Hz), 0.91 (1H, *dd*, *J* = 3.8, 2.1 Hz), 0.89 (3H, *s*, H-15), 0.83 (1H, *td*, *J* = 8.4, 6.0 Hz). ^13^C NMR (125 MHz, chloro­form-*d*): δ 171.34 (OCOCH_3_ or C-12), 171.31 (OCOCH_3_ or C-12), 149.69 (C-8), 148.41 (C-7), 122.47 (C-11), 120.13 (C-9), 66.23 (C-15), 60.45 (C-5), 43.11 (C-4), 42.15 (C-10), 27.47 (C-1), 22.87 (C-6), 22.48 (C-3), 21.25 (OCOCH_3_ or C-14), 21.21 (OCOCH_3_ or C-14), 17.15 (C-2), 8.83 (C-13).

## 7. Refinement

Crystal data, data collection and structure refinement details are summarized in Table 2 ▸. All H atoms were positioned geometrically (C—H = 0.96–0.98 Å) and refined as riding, with *U*_iso_(H) = 1.2*U*_eq_(C) for CH hydrogens or 1.5*U*_eq_(C) for methyl H atoms.

## Supplementary Material

Crystal structure: contains datablock(s) I, global. DOI: 10.1107/S2056989022004625/zn2018sup1.cif

Structure factors: contains datablock(s) I. DOI: 10.1107/S2056989022004625/zn2018Isup2.hkl

Supporting information file. DOI: 10.1107/S2056989022004625/zn2018Isup3.cml

CCDC reference: 2169817

Additional supporting information:  crystallographic information; 3D view; checkCIF report

## Figures and Tables

**Table 1:** Hydrogen-bond geometry (Å, °)

*D*—H⋯*A*	*D*—H	H⋯*A*	*D*⋯*A*	*D*—H⋯*A*
C8—H8*A*⋯O1^i^	0.97	2.81	3.481 (5)	127
C11—H11⋯O3^ii^	0.98	2.54	3.497 (5)	167
C13—H13*C*⋯O1^iii^	0.96	2.60	3.499 (5)	157
C13—H13*B*⋯O4^iv^	0.96	2.61	3.530 (6)	160
C14—H14*A*⋯O2^i^	0.96	2.76	3.530 (5)	138
C17—H17*C*⋯O3^v^	0.96	2.86	3.478 (5)	124

Symmetry codes: (i) 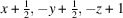 ; (ii) 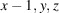 ; (iii) 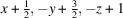 ; (iv) 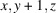 ; (v) 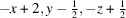 .

**Table 2:** Experimental details

Crystal data	
Chemical formula	C_17_H_20_O_4_
*M*_r_	288.33
Crystal system, space group	Orthorhombic, *P*2_1_2_1_2_1_
Temperature (K)	296
*a*, *b*, *c* (Å)	6.7641 (3), 6.9254 (3), 31.4538 (14)
*V* (Å^3^)	1473.42 (11)
*Z*	4
Radiation type	Mo *K*α
μ (mm^−1^)	0.09
Crystal size (mm)	0.20 × 0.20 × 0.20

Data collection	
Diffractometer	Bruker SMART CCD
Absorption correction	Multi-scan (*SADABS*; Sheldrick, 1996 ▸)
No. of measured, independent and observed [*I* > 2σ(*I*)] reflections	12659, 2576, 1857
*R*_int_	0.057
(sin θ/λ)_max_ (Å^−1^)	0.595

Refinement	
*R*[*F* ^2^ > 2σ(*F* ^2^)], *wR*(*F* ^2^), *S*	0.051, 0.117, 1.05
No. of reflections	2576
No. of parameters	193
H-atom treatment	H-atom parameters constrained
Δρ_max_, Δρ_min_ (e Å^−3^)	0.30, −0.21
Absolute structure	Flack *x* determined using 574 quotients [(*I* ^+^)−(*I* ^−^)]/[(*I* ^+^)+(*I* ^−^)] (Parsons *et al.*, 2013 ▸)
Absolute structure parameter	0.10 (8)

Computer programs: *SMART* and *SAINT* (Bruker, 2002 ▸), *SHELXT2014* (Sheldrick, 2015*a* ▸), *SHELXL2014* (Sheldrick, 2015*b* ▸), *OLEX2* (Dolomanov *et al.*, 2009 ▸) and *publCIF* (Westrip, 2010 ▸).

## supplementary crystallographic information

### Crystal data

**Table:**

C_17_H_20_O_4_	*D*_x_ = 1.300 Mg m^−^^3^
*M**_r_* = 288.33	Mo *K*α radiation, λ = 0.71073 Å
Orthorhombic, *P*2_1_2_1_2_1_	Cell parameters from 3545 reflections
*a* = 6.7641 (3) Å	θ = 2.6–20.7°
*b* = 6.9254 (3) Å	µ = 0.09 mm^−^^1^
*c* = 31.4538 (14) Å	*T* = 296 K
*V* = 1473.42 (11) Å^3^	Block, colorless
*Z* = 4	0.20 × 0.20 × 0.20 mm
*F*(000) = 616	

### Data collection

**Table:**

Bruker SMART CCD diffractometer	1857 reflections with *I* > 2σ(*I*)
phi and ω scans	*R*_int_ = 0.057
Absorption correction: multi-scan (SADABS; Sheldrick, 1996)	θ_max_ = 25.0°, θ_min_ = 2.6°
	*h* = −7→8
12659 measured reflections	*k* = −8→6
2576 independent reflections	*l* = −32→37

### Refinement

**Table:**

Refinement on *F*^2^	Hydrogen site location: inferred from neighbouring sites
Least-squares matrix: full	H-atom parameters constrained
*R*[*F*^2^ > 2σ(*F*^2^)] = 0.051	*w* = 1/[σ^2^(*F*_o_^2^) + (0.0466*P*)^2^ + 0.380*P*] where *P* = (*F*_o_^2^ + 2*F*_c_^2^)/3
*wR*(*F*^2^) = 0.117	(Δ/σ)_max_ < 0.001
*S* = 1.05	Δρ_max_ = 0.30 e Å^−^^3^
2576 reflections	Δρ_min_ = −0.21 e Å^−^^3^
193 parameters	Absolute structure: Flack *x* determined using 574 quotients [(I+)-(I-)]/[(I+)+(I-)] (Parsons *et al.*, 2013)
0 restraints	Absolute structure parameter: 0.10 (8)

### Special details

**Table:**

Geometry. All esds (except the esd in the dihedral angle between two l.s. planes) are estimated using the full covariance matrix. The cell esds are taken into account individually in the estimation of esds in distances, angles and torsion angles; correlations between esds in cell parameters are only used when they are defined by crystal symmetry. An approximate (isotropic) treatment of cell esds is used for estimating esds involving l.s. planes.

### Fractional atomic coordinates and isotropic or equivalent isotropic displacement parameters (Å^2^)

**Table:**

	*x*	*y*	*z*	*U*_iso_*/*U*_eq_	
O1	0.1658 (5)	0.5144 (4)	0.54793 (8)	0.0745 (9)	
O2	0.1329 (4)	0.5303 (4)	0.47683 (7)	0.0537 (7)	
O3	0.9151 (4)	0.1520 (4)	0.31668 (8)	0.0566 (8)	
O4	0.9315 (6)	−0.1600 (5)	0.32844 (14)	0.1261 (18)	
C1	0.2446 (6)	0.5140 (6)	0.51366 (12)	0.0536 (10)	
C2	0.4539 (6)	0.4960 (5)	0.50214 (10)	0.0480 (9)	
C3	0.4648 (5)	0.4991 (5)	0.45952 (10)	0.0418 (8)	
C4	0.2650 (5)	0.5215 (5)	0.44282 (11)	0.0442 (9)	
C5	0.2143 (5)	0.5386 (5)	0.40250 (11)	0.0447 (10)	
H5	0.0829	0.5495	0.3942	0.054*	
C6	0.3810 (5)	0.5396 (5)	0.37059 (10)	0.0389 (9)	
C7	0.5465 (5)	0.4057 (5)	0.38746 (10)	0.0387 (9)	
H7	0.4802	0.2839	0.3944	0.046*	
C8	0.6345 (5)	0.4765 (6)	0.42937 (10)	0.0426 (9)	
H8A	0.7290	0.3836	0.4403	0.051*	
H8B	0.7014	0.5991	0.4253	0.051*	
C9	0.6749 (5)	0.3603 (5)	0.34836 (10)	0.0414 (9)	
H9	0.7586	0.4713	0.3414	0.050*	
C10	0.5169 (6)	0.3322 (6)	0.31409 (11)	0.0503 (10)	
H10	0.4988	0.2025	0.3023	0.060*	
C11	0.3340 (6)	0.4435 (6)	0.32784 (11)	0.0506 (11)	
H11	0.2047	0.3818	0.3243	0.061*	
C12	0.4461 (6)	0.5003 (7)	0.28864 (10)	0.0613 (11)	
H12A	0.3853	0.4739	0.2613	0.074*	
H12B	0.5235	0.6181	0.2897	0.074*	
C13	0.4477 (6)	0.7496 (5)	0.36559 (12)	0.0523 (11)	
H13A	0.3469	0.8217	0.3511	0.078*	
H13B	0.5679	0.7539	0.3494	0.078*	
H13C	0.4701	0.8050	0.3932	0.078*	
C14	0.6127 (7)	0.4764 (6)	0.53450 (12)	0.0650 (12)	
H14A	0.6047	0.3512	0.5475	0.098*	
H14B	0.5968	0.5743	0.5558	0.098*	
H14C	0.7393	0.4911	0.5211	0.098*	
C15	0.8003 (6)	0.1832 (5)	0.35511 (11)	0.0488 (10)	
H15A	0.8877	0.2019	0.3792	0.059*	
H15B	0.7171	0.0720	0.3608	0.059*	
C16	0.9745 (5)	−0.0230 (6)	0.30723 (13)	0.0550 (10)	
C17	1.1017 (6)	−0.0305 (7)	0.26893 (12)	0.0660 (12)	
H17A	1.2179	0.0462	0.2735	0.099*	
H17B	1.0301	0.0192	0.2450	0.099*	
H17C	1.1394	−0.1619	0.2635	0.099*	

### Atomic displacement parameters (Å^2^)

**Table:**

	*U*^11^	*U*^22^	*U*^33^	*U*^12^	*U*^13^	*U*^23^
O1	0.106 (2)	0.074 (2)	0.0437 (16)	0.017 (2)	0.0283 (16)	−0.0019 (15)
O2	0.0592 (16)	0.0591 (17)	0.0427 (15)	0.0035 (15)	0.0182 (13)	−0.0021 (13)
O3	0.076 (2)	0.0422 (16)	0.0520 (16)	0.0031 (15)	0.0267 (16)	−0.0038 (13)
O4	0.137 (4)	0.058 (2)	0.184 (4)	0.013 (2)	0.101 (3)	0.014 (3)
C1	0.080 (3)	0.040 (2)	0.040 (2)	0.013 (2)	0.014 (2)	−0.001 (2)
C2	0.069 (3)	0.036 (2)	0.039 (2)	0.002 (2)	0.0037 (19)	−0.0026 (18)
C3	0.054 (2)	0.0344 (19)	0.0366 (19)	0.000 (2)	0.0037 (17)	−0.0007 (17)
C4	0.049 (2)	0.043 (2)	0.040 (2)	0.000 (2)	0.0113 (18)	−0.0030 (19)
C5	0.038 (2)	0.052 (2)	0.044 (2)	−0.003 (2)	0.0027 (17)	−0.0035 (19)
C6	0.0395 (19)	0.045 (2)	0.0324 (18)	−0.0053 (18)	0.0005 (16)	−0.0023 (16)
C7	0.042 (2)	0.040 (2)	0.0340 (19)	−0.0054 (18)	0.0046 (17)	−0.0033 (15)
C8	0.044 (2)	0.047 (2)	0.0366 (18)	−0.003 (2)	−0.0007 (16)	−0.0045 (17)
C9	0.045 (2)	0.041 (2)	0.039 (2)	−0.0055 (18)	0.0052 (18)	−0.0040 (16)
C10	0.054 (2)	0.058 (3)	0.039 (2)	−0.005 (2)	0.005 (2)	−0.0120 (19)
C11	0.044 (2)	0.068 (3)	0.040 (2)	−0.011 (2)	0.0019 (18)	−0.0067 (18)
C12	0.061 (2)	0.091 (3)	0.0322 (19)	−0.002 (3)	−0.0002 (18)	0.002 (2)
C13	0.057 (3)	0.048 (2)	0.052 (2)	−0.006 (2)	0.000 (2)	0.0053 (18)
C14	0.093 (3)	0.058 (3)	0.044 (2)	0.004 (3)	−0.006 (2)	−0.003 (2)
C15	0.062 (2)	0.046 (2)	0.039 (2)	−0.001 (2)	0.015 (2)	−0.0029 (18)
C16	0.047 (2)	0.048 (3)	0.070 (3)	−0.006 (2)	0.013 (2)	−0.007 (2)
C17	0.063 (3)	0.071 (3)	0.064 (3)	0.006 (3)	0.014 (2)	−0.017 (2)

### Geometric parameters (Å, º)

**Table:**

O1—C1	1.202 (4)	C9—C15	1.507 (5)
O2—C1	1.388 (4)	C9—C10	1.530 (5)
O2—C4	1.396 (4)	C9—H9	0.9800
O3—C16	1.311 (5)	C10—C12	1.492 (6)
O3—C15	1.453 (4)	C10—C11	1.520 (5)
O4—C16	1.196 (5)	C10—H10	0.9800
C1—C2	1.467 (6)	C11—C12	1.500 (5)
C2—C3	1.343 (4)	C11—H11	0.9800
C2—C14	1.486 (5)	C12—H12A	0.9700
C3—C4	1.458 (5)	C12—H12B	0.9700
C3—C8	1.497 (5)	C13—H13A	0.9600
C4—C5	1.319 (5)	C13—H13B	0.9600
C5—C6	1.509 (5)	C13—H13C	0.9600
C5—H5	0.9300	C14—H14A	0.9600
C6—C13	1.531 (5)	C14—H14B	0.9600
C6—C11	1.534 (5)	C14—H14C	0.9600
C6—C7	1.548 (5)	C15—H15A	0.9700
C7—C8	1.528 (4)	C15—H15B	0.9700
C7—C9	1.538 (5)	C16—C17	1.481 (5)
C7—H7	0.9800	C17—H17A	0.9600
C8—H8A	0.9700	C17—H17B	0.9600
C8—H8B	0.9700	C17—H17C	0.9600
			
C1—O2—C4	106.7 (3)	C11—C10—C9	107.7 (3)
C16—O3—C15	119.3 (3)	C12—C10—H10	118.1
O1—C1—O2	120.4 (4)	C11—C10—H10	118.1
O1—C1—C2	130.5 (4)	C9—C10—H10	118.1
O2—C1—C2	109.0 (3)	C12—C11—C10	59.2 (3)
C3—C2—C1	107.4 (3)	C12—C11—C6	120.1 (3)
C3—C2—C14	130.2 (4)	C10—C11—C6	107.5 (3)
C1—C2—C14	122.4 (3)	C12—C11—H11	118.2
C2—C3—C4	108.1 (3)	C10—C11—H11	118.2
C2—C3—C8	132.3 (3)	C6—C11—H11	118.2
C4—C3—C8	119.6 (3)	C10—C12—C11	61.1 (3)
C5—C4—O2	124.5 (3)	C10—C12—H12A	117.7
C5—C4—C3	126.6 (3)	C11—C12—H12A	117.7
O2—C4—C3	108.8 (3)	C10—C12—H12B	117.7
C4—C5—C6	116.5 (3)	C11—C12—H12B	117.7
C4—C5—H5	121.8	H12A—C12—H12B	114.8
C6—C5—H5	121.8	C6—C13—H13A	109.5
C5—C6—C13	107.0 (3)	C6—C13—H13B	109.5
C5—C6—C11	115.2 (3)	H13A—C13—H13B	109.5
C13—C6—C11	112.5 (3)	C6—C13—H13C	109.5
C5—C6—C7	108.0 (3)	H13A—C13—H13C	109.5
C13—C6—C7	113.1 (3)	H13B—C13—H13C	109.5
C11—C6—C7	101.0 (3)	C2—C14—H14A	109.5
C8—C7—C9	122.4 (3)	C2—C14—H14B	109.5
C8—C7—C6	112.7 (3)	H14A—C14—H14B	109.5
C9—C7—C6	104.9 (3)	C2—C14—H14C	109.5
C8—C7—H7	105.2	H14A—C14—H14C	109.5
C9—C7—H7	105.2	H14B—C14—H14C	109.5
C6—C7—H7	105.2	O3—C15—C9	107.7 (3)
C3—C8—C7	106.3 (3)	O3—C15—H15A	110.2
C3—C8—H8A	110.5	C9—C15—H15A	110.2
C7—C8—H8A	110.5	O3—C15—H15B	110.2
C3—C8—H8B	110.5	C9—C15—H15B	110.2
C7—C8—H8B	110.5	H15A—C15—H15B	108.5
H8A—C8—H8B	108.7	O4—C16—O3	122.2 (4)
C15—C9—C10	112.9 (3)	O4—C16—C17	124.5 (4)
C15—C9—C7	111.8 (3)	O3—C16—C17	113.3 (4)
C10—C9—C7	101.2 (3)	C16—C17—H17A	109.5
C15—C9—H9	110.2	C16—C17—H17B	109.5
C10—C9—H9	110.2	H17A—C17—H17B	109.5
C7—C9—H9	110.2	C16—C17—H17C	109.5
C12—C10—C11	59.7 (3)	H17A—C17—H17C	109.5
C12—C10—C9	120.2 (4)	H17B—C17—H17C	109.5
			
C4—O2—C1—O1	−178.9 (4)	C4—C3—C8—C7	−21.5 (5)
C4—O2—C1—C2	0.5 (4)	C9—C7—C8—C3	−178.6 (3)
O1—C1—C2—C3	178.5 (4)	C6—C7—C8—C3	55.0 (4)
O2—C1—C2—C3	−0.8 (5)	C8—C7—C9—C15	69.2 (4)
O1—C1—C2—C14	−0.9 (7)	C6—C7—C9—C15	−161.0 (3)
O2—C1—C2—C14	179.8 (3)	C8—C7—C9—C10	−170.4 (3)
C1—C2—C3—C4	0.7 (5)	C6—C7—C9—C10	−40.5 (3)
C14—C2—C3—C4	−179.9 (4)	C15—C9—C10—C12	−151.0 (3)
C1—C2—C3—C8	−176.5 (4)	C7—C9—C10—C12	89.3 (4)
C14—C2—C3—C8	2.9 (7)	C15—C9—C10—C11	144.3 (3)
C1—O2—C4—C5	−177.6 (4)	C7—C9—C10—C11	24.6 (4)
C1—O2—C4—C3	0.0 (4)	C9—C10—C11—C12	115.2 (4)
C2—C3—C4—C5	177.1 (4)	C12—C10—C11—C6	−115.2 (4)
C8—C3—C4—C5	−5.3 (6)	C9—C10—C11—C6	0.0 (4)
C2—C3—C4—O2	−0.4 (4)	C5—C6—C11—C12	155.4 (4)
C8—C3—C4—O2	177.2 (3)	C13—C6—C11—C12	32.3 (5)
O2—C4—C5—C6	175.6 (3)	C7—C6—C11—C12	−88.5 (4)
C3—C4—C5—C6	−1.5 (6)	C5—C6—C11—C10	−140.6 (3)
C4—C5—C6—C13	−88.5 (4)	C13—C6—C11—C10	96.4 (4)
C4—C5—C6—C11	145.6 (4)	C7—C6—C11—C10	−24.5 (4)
C4—C5—C6—C7	33.5 (4)	C9—C10—C12—C11	−93.8 (4)
C5—C6—C7—C8	−62.8 (4)	C6—C11—C12—C10	93.4 (4)
C13—C6—C7—C8	55.4 (4)	C16—O3—C15—C9	−153.7 (3)
C11—C6—C7—C8	175.8 (3)	C10—C9—C15—O3	66.5 (4)
C5—C6—C7—C9	161.8 (3)	C7—C9—C15—O3	179.9 (3)
C13—C6—C7—C9	−80.0 (3)	C15—O3—C16—O4	2.8 (6)
C11—C6—C7—C9	40.5 (3)	C15—O3—C16—C17	−175.9 (3)
C2—C3—C8—C7	155.5 (4)		

### Hydrogen-bond geometry (Å, º)

**Table:**

*D*—H···*A*	*D*—H	H···*A*	*D*···*A*	*D*—H···*A*
C8—H8*A*···O1^i^	0.97	2.81	3.481 (5)	127
C11—H11···O3^ii^	0.98	2.54	3.497 (5)	167
C13—H13*C*···O1^iii^	0.96	2.60	3.499 (5)	157
C13—H13*B*···O4^iv^	0.96	2.61	3.530 (6)	160
C14—H14*A*···O2^i^	0.96	2.76	3.530 (5)	138
C17—H17*C*···O3^v^	0.96	2.86	3.478 (5)	124

Symmetry codes: (i) *x*+1/2, −*y*+1/2, −*z*+1; (ii) *x*−1, *y*, *z*; (iii) *x*+1/2, −*y*+3/2, −*z*+1; (iv) *x*, *y*+1, *z*; (v) −*x*+2, *y*−1/2, −*z*+1/2.
